# *Pneumocystis Jirovecii* Pneumonia after Initiation of Tofacitinib Therapy in Rheumatoid Arthritis: Case-Based Review

**DOI:** 10.31138/mjr.30.3.167

**Published:** 2019-09-30

**Authors:** Ioannis Grigoropoulos, Konstantinos Thomas, Panagiotis Christoforou, Iliana Fanidi, Maria Papavdi, Fani Kyriakou, Melanie Deutsch, Maria Pirounaki, Dimitrios Vassilopoulos

**Affiliations:** 2^nd^ Department of Medicine and Laboratory, National and Kapodistrian University of Athens Medical School, Hippokration General Hospital, Athens, Greece

**Keywords:** rheumatoid arthritis, tofacitinib, *Pneumocystis jirovecii*, pneumonia

## Abstract

The use of biologic and targeted synthetic disease-modifying anti-rheumatic drugs (tsDMARDs) in rheumatic diseases is constantly increasing during the last decade. Tofacitinib is a new oral Janus Kinase (JAK) inhibitor, approved for rheumatoid arthritis (RA), psoriatic arthritis and ulcerative colitis. Safety data of tofacitinib derived from randomized controlled trials and long-term extension studies has demonstrated a moderate increase in the risk for common serious infections. We describe a case of *Pneumocystis jirovecii* pneumonia (PJP) in a woman on tofacitinib therapy for RA. Although tofacitinib use has been associated with the development of opportunistic infections, PJP has been rarely reported. PJP should be included in the differential diagnosis of patients with autoimmune disorders under newer oral JAK inhibitors therapy who present with fever, hypoxia and pulmonary infiltrates.

## INTRODUCTION

Tofacitinib is a novel oral Janus kinase (JAK) inhibitor which inhibits the production of various pro-inflammatory cytokines by T-lymphocytes and dendritic cells.^1^ It has been already approved for the treatment of rheumatoid arthritis (RA), psoriatic arthritis and ulcerative colitis.^1^ *Pneumocystis jirovecii* pneumonia (PJP) is a relatively rare opportunistic infection in patients with rheumatic diseases under immunosuppressive therapy.

Here we discuss an interesting case of PJP in a patient with RA treated with tofacitinib. We also performed a literature review in MEDLINE, using the search terms “tofacitinib” OR “baricitinib” AND “Pneumocystis”. No language, publication date or publication status restrictions were imposed. We identified one case-report, one combined phase II, III and long-term extension (LTE) study and two post-marketing surveillance (PMS) reports (one published manuscript and one conference abstract) of RA patients treated with tofacitinib. Regarding baricitinib, we identified one conference abstract reporting detailed safety data from phase Ib, II, III and LTE studies in patients with RA.

## CASE DESCRIPTION

A 79-year-old female presented with a 5-day history of fever (up to 39°C), non-productive cough and shortness of breath, not responding to an outpatient course of antibiotics (moxifloxacin followed by cefixime and azithromycin) and oseltamivir.

The patient had a 10-year history of RA for which she had been treated with various non-biologic (methotrexate) and biologic (including Tumor Necrosis Factor-Inhibitors-TNFi and abatacept) disease-modifying anti-rheumatic drugs (DMARDs), without an adequate response. Two months before presentation, tofacitinib (5 mg per os twice a day) was added to her therapy, which included methotrexate (15 mg per os once a week) and prednisolone (5 mg/day per os). Her past medical history was significant for arterial hypertension and hyperlipidaemia, while she had been vaccinated against pneumococcus and influenza.

On presentation, she was tachypnoeic (28 breaths/min) and febrile (38.2°C) with bilateral expiratory wheezing. The oxygen saturation was 90% while she was breathing ambient air. Her initial chest x-ray was unremarkable, but a chest CT showed diffuse ground glass opacities, especially in the upper lobes. Laboratory data on admission revealed hyponatremia (132 mEq/L), mildly elevated LDH (292 U/L, normal <220 U/L), elevated CRP (88 mg/L, < 5 mg/L) and lymphopenia (WBC= 8,710/μL, absolute lymphocyte count = 700/μL).

Broad-spectrum antibiotics (meropenem and levofloxacin) and oseltamivir were administered, but despite treatment the patient remained febrile without improvement of hypoxia. Induced sputum samples tested for *Influenza, Parainfluenza, RSV, Adenovirus, CMV, Mycoplasma and Chlamydophila* (by PCR), as well as for acid-fast bacilli (specific stains, PCR) were negative. PCR in induced sputum was positive for *Pneumocystis jirovecii* and, along with the compatible clinical presentation and imaging findings, pointed to a diagnosis of PJP.

**Figure 1.**
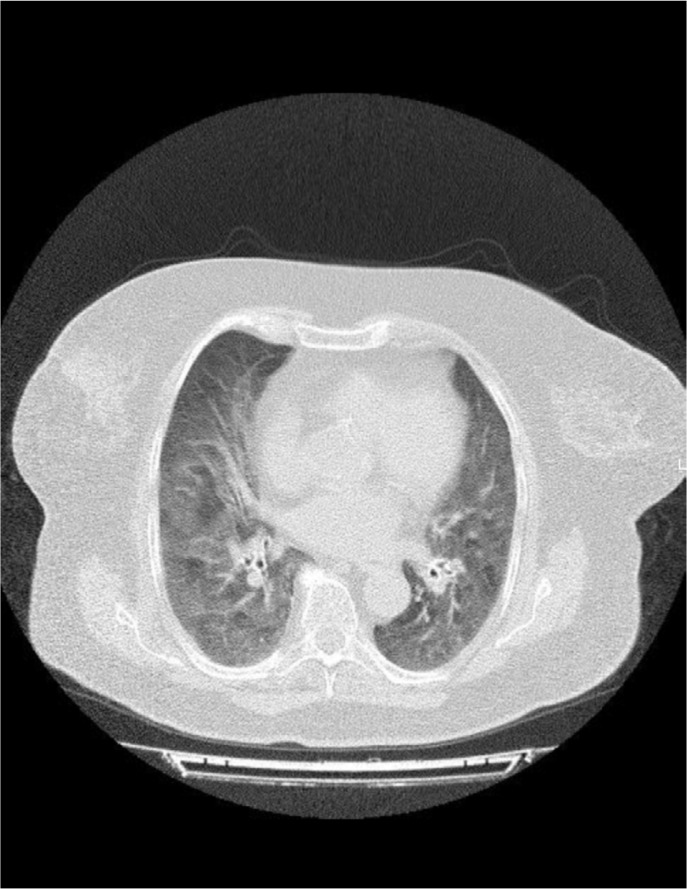
Chest computed tomography at admission showing bilateral ground glass opacities.

Her therapy was switched to trimethoprim/sulfamethoxazole (TMP/SMX, 15mg TMP/kg/day) and prednisolone (1 mg/kg/day) with significant clinical improvement and resolution of hypoxia. Fifteen days later, TMP/SMX was stopped due to agranulocytosis (WBC=1,970/μL, Neutrophils=150/μL) and she was started on primaquine-clindamycin along with filgrastim. Gradually, her agranulocytosis resolved and she was discharged home after completion of a 21-day treatment course. Subsequently, oral atovaquone was prescribed as secondary PJP prophylaxis for 6 months.

## DISCUSSION

Oral JAK inhibitors are being used for an expanding variety of indications including RA and more recently psoriatic arthritis and ulcerative colitis.^1^ For RA, tofacitinib and baricitinib have been approved and are recommended after failure of non-biologic (methotrexate, leflunomide) or biologic (TNFi, anti-interleukin (IL)-6, abatacept, anti-B cell-rituximab) agents.^1,2^

Safety data of tofacitinib from randomized controlled trials and their long-term extension studies have demonstrated a moderate increase in the risk for common serious bacterial infections when compared to csDMARDs (2.7/100 patient-years), similar to bDMARDs.^3^ Factors that contribute to increased risk include older age, diabetes, corticosteroid use, high dose of tofacitinib (10 mg twice a day) and lymphopenia (<500/μL).^3^

PJP is a fungal respiratory infection more common in HIV and heavily immunosuppressed (for hematologic and solid organ malignancies, systemic rheumatic diseases) patients. Regarding rheumatic patients, risk factors for PJP include an underlying diagnosis of ANCA-associated vasculitis or inflammatory myopathy, high-dose corticosteroid therapy, lymphopenia and treatment with cyclophosphamide or rituximab.^4,5^ Of note, our patient had none of these aforementioned risk factors.

In general, RA patients are not considered a population at risk for PJP, regardless of treatment type. As for TNFi, the majority of cases were reported in Southeast Asia and additional risk factors were recognized (older age, chronic lung disease, diabetes, high-dose corticosteroids).^6^ No association between non-TNFi biologic agents and PJP has been established.

There already is adequate clinical experience with tofacitinib so far and, although opportunistic infections such as herpes zoster, invasive fungal infections and tuberculosis (especially in highly endemic areas) have been reported, PJP has been rarely recorded.^7^ Only 5 PJP cases were identified among 6,194 RA patients (19,406 patient-years of follow-up) treated in Phase I, II, III and their long-term extension studies,^3^ whereas in a worldwide 3-year PMS study of tofacitinib in RA patients, there was only 1 case of PJP (∼35,000 patient-years of follow-up).^8^ An interim analysis of PMS data in Japanese patients reported 12 cases of PJP during 1,704 patient-years of follow-up, a significantly higher incidence than the one reported worldwide.^9^ Interestingly, during the Global Clinical Trials of tofacitinib in patients with ulcerative colitis (median treatment duration: 1.4 years, total follow-up: 1,613 patient-years), no case of PJP was reported.^10^ Baricitinib is a different drug belonging in the same class of JAK inhibitors and is currently approved for treatment of moderate to severe RA. Winthrop et al reported only 3 cases of PJP during 6,637 patient-years of follow-up in the baricitinib program trials.^11^

To our knowledge, this is the second reported case of PJP in tofacitinib-treated RA patients outside of the clinical trials or post-marketing surveillance context. Our case shares many similarities to the one recently published by Pirker et al.^12^ Both patients had started tofacitinib recently (2 months before presentation) along with methotrexate and low dose prednisolone. This temporal correlation may be of interest, indicating a higher risk for PJP during the first months of treatment, as has been shown for other treatments, such as with TNFi.

The diagnosis of PJP was established by a positive PCR in induced sputum in our case and in bronchoalveolar lavage (BAL) in the other case.^12^ During the last decade the diagnostic role of PCR for *Pneumocystis jirovecii* isolation in respiratory specimens has been expanded, being more sensitive than conventional stains or immunofluorescence techniques. Recently, Doyle et al. estimated its positive predictive value at 93%, proposing it as the diagnostic test of choice.^13^ Concerning the specimens tested, BAL fluid is the preferred one, while induced sputum is considered an acceptable alternative. Nevertheless, in contrast to BAL PCR, a negative induced sputum PCR cannot definitely rule out PJP.^14^ Conclusively, a positive PCR (either in BAL or induced sputum) for *Pneumocystis jirovecii* combined with consistent clinical and radiological findings sets the diagnosis of PJP, as in both reported cases.

In contrast to the case report from Pirker et al., our patient had a typical non-HIV clinical presentation of PJP with fever, cough and shortness of breath. Lastly, both cases improved with TMP/SMX therapy, but had to switch to second-line regimens due to TMP/SMX-related adverse events (agranulocytosis in our case and hyponatremia in the other case).^12^

There are no consensus guidelines for the treatment and prophylaxis of PJP in RA patients under immunosuppressive therapy. Recommendations are based on studies regarding PJP in HIV patients and observational studies in non-HIV patients with iatrogenic immunosuppression. TMP/SMX is recommended as the first-line treatment of mild to severe PJP.^15^ Alternative regimens when TMP/SMX cannot be used include clindamycin plus primaquine, trimethoprim plus dapsone, atovaquone or intravenous pentamidine. The choice of regimen depends upon patient’s profile and the severity of disease. Adjunctive glucocorticoids in the treatment of PJP in non-HIV patients is commonly used, although the available data in this population is limited and conflicting. Optimal duration of therapy is not well established, however evidence extrapolated from HIV patients with PJP suggest that 21-day therapy is more effective than shorter duration regimens,^15^ regarding the risk of relapse. Primary prophylaxis for the prevention of PJP in the non-HIV population is recommended when the incidence of PJP exceeds 3.5%^16^ and, as previously mentioned, high-risk rheumatic patients are regarded those with ANCA-associated vasculitis or inflammatory myopathy, receiving high doses of corticosteroids (> 15 mg prednisolone daily), those with baseline lymphopenia or low CD4 counts, concomitant use of cyclophosphamide, TNFi or rituximab and initial glucocorticoid dose of > 60mg of prednisolone.^4,5^ Primary prophylaxis with TMP/SMX almost eliminates the risk of PJP^4^ and is considered the first-line agent. The proportion of patients that develop adverse events is approximately 15%, although only 3.1% discontinue TMP/SMX.^17^ In a recent study from South Korea, the incidence of TMP/SMX-related adverse events was 21.2/100 patient-years, but only 2 cases (1.2/100 patient-years) were considered serious.^4^ Other options for primary prophylaxis (atovaquone, dapsone with or without pyrimethamine and aerosolized pentamidine) are not well studied in this particular context. Based on recent expert opinion, duration of prophylaxis should be individualized taking into account the underlying diagnosis, corticosteroid dose and other risk factors.^5^ Concerning secondary prophylaxis, there is limited data in the rheumatic population, although older case series reported no relapse of PJP, even after reinstitution of immunosuppression.^18^

## CONCLUSION

Although rare, PJP is a potentially fatal infection if not recognized early and should be included in the differential diagnosis of patients presenting with fever, hypoxia and pulmonary infiltrates under treatment with JAK inhibitors (especially during the first months of treatment). When PJP is suspected, prompt diagnosis and initiation of specific therapy are crucial for a favourable outcome.
